# Microwave-assisted synthesis of 2’-hydroxychalcone derivatives in 1-decyl-3-methylimidazolium bromide ionic liquid and its computational study

**DOI:** 10.1098/rsos.251265

**Published:** 2025-11-26

**Authors:** Prisma Silviya Auliawati, Atthar Luqman Ivansyah, Deana Wahyuningrum

**Affiliations:** ^1^ Department of Chemistry, Institut Teknologi Bandung Fakultas Matematika dan Ilmu Pengetahuan Alam, Bandung, Jawa Barat, Indonesia

**Keywords:** microwave-assisted synthesis, hydroxychalcone, ionic liquid, computational study

## Abstract

Three 2’-hydroxychalcone derivatives were synthesized via microwave-assisted organic synthesis using the ionic liquid 1-decyl-3-methylimidazolium bromide ([DMIm]Br) as the medium. The reactions, conducted at 80°C under 300 W microwave power for 10 min, yielded products with yields of 65, 72 and 81%. A computational study using density functional theory with B3LYP and ωB97X functionals examined the reactivity of precursors via Fukui function analysis. Results showed that higher reactivity of benzaldehyde derivatives correlated with increased product yields: *o*-vanillin > anisaldehyde > salicylaldehyde. The cyclization of 2,2’-dihydroxy-3-methoxychalcone to its flavanone derivative was also simulated, revealing a two-step mechanism with the first step being rate-determining (activation energy: 63.8 kJ mol^−1^). Additionally, the formation of [DMIm]Br was found to proceed through an S_N_2 mechanism, with an activation energy of 197.3  kJ mol^−1^. These experimental and computational findings underscore the predictive power of Fukui-based reactivity in optimizing chalcone synthesis in ionic liquid media.

## 1. Introduction

Ionic liquids are ionic salts whose cations are organic compounds. At the same time, the anions can be organic, inorganic compounds or a combination of both which have the characteristics of low vapour pressure and thermal stability which is limited by the strength of their heteroatomic bonds, the ability to dissolve various compounds, good electrochemical stability, not flammable and can be applied as a catalyst that can speed up chemical reactions [1]. Ionic liquids are widely used as a medium in various organic reactions because of their environmentally friendly nature [2,3].

Hydrogen bonds play a crucial role in the ability of ionic liquids to dissolve materials that are insoluble in other media [4]. One application of ionic liquids is as a reaction medium in the synthesis of chalcone derivative compounds, which constitute the majority subgroup of the flavonoid secondary metabolite class, which has been reported to have various biological activities because it is considered a class with important therapeutic potential [5,6], such as antibacterial [7,8], anti-cancer [9–11], antiviral [12–14], anti-inflammatory [15,16] and anti-oxidant [17–19].

Chalcone derivative compounds are synthesized via the Claisen–Schmidt condensation reaction, which involves cross-aldol condensation between aromatic aldehyde and aromatic ketone derivatives using a base or acid catalyst, followed by dehydration to form unsaturated bonds [20]. The base catalysts commonly used in chalcone synthesis include LiOH, NaOH, KOH and sodium ethoxide [21].

The synthesis of chalcone derivative compounds has been carried out previously using various methods. The conventional method for synthesizing 2,2’-dihydroxychalcone involves using a NaOH catalyst in an ethanol solvent with a reflux method at 30°C for 24 hours, yielding 15.6% of the product. Additionally, the synthesis of 2’-hydroxy-4-methoxychalcone was also carried out under the same conditions, yielding 40.3% of the product [22]. Another study reported that the compound 2,3’-dihydroxy-3-methoxychalcone was synthesized using two equivalents of NaOH as a catalyst at a temperature of 70°C for 3 hours, yielding a 40% yield [23].

The synthesis of chalcones in the presence of various ionic liquids has been previously reported. Ionic liquids have been proven to be catalysts that can accelerate the reaction; however, with conventional heating methods, the reaction takes 4 hours [24]. Synthesis using conventional methods is known to require a relatively long reaction time, so non-conventional methods are needed that can shorten this time.

Synthesis assisted by microwave (MW) irradiation can lead to a considerable increase in the reaction rate and produce better products. Heating with MW irradiation directly affects all the molecules involved in the reaction mixture, causing a faster temperature increase that enables the reaction to proceed more quickly [24]. In synthetic applications using MW irradiation, the choice of solvent as a reaction medium is critical. MW heating is particularly suitable for solvents such as water, ionic liquids or alcohols, which are environmentally friendly [25].

Chalcone is also the precursor of flavonoids and isoflavonoids, which are abundant in various plants and can be transformed into flavanone group compounds through a transformation using acids, bases and organic bases as catalysts. The conversion of 2’-hydroxychalcone to flavanone involves a 6-endo-trig cyclization mechanism, and in biological systems, this reaction is catalysed by the enzyme chalcone isomerase. In the laboratory, this transformation is carried out using acids, bases and organic bases as catalysts [26].

In-depth studies of this group of compounds are needed, considering their high potential in treating various diseases. Computational studies can be conducted using the density functional theory (DFT) method, a quantum mechanical modelling approach that determines the electronic structure of many-electron systems. Formulated in terms of electron density, the DFT method can be applied to calculate the Schrödinger equation for many-particle systems [27]. This study was conducted to support the results of previous experiments and to predict and plan the synthesis that will be undertaken.

## 2. Material and methods

### 2.1. Materials

1-methylimidazole (Sigma Aldrich), *n*-bromodecane (Sigma Aldrich), anisaldehyde (Sigma Aldrich), salicylaldehyde (Sigma Aldrich), *ortho*-hydroxyacetophenone (Sigma Aldrich), NaOH 40% (w/v), *n*-hexane p.a (Merck), chloroform p.a (Merck), dichloromethane p.a (Merck), ethyl acetate p.a (Merck), ethanol p.a (Merck) and methanol p.a (Merck) were used for thin-layer chromatography (TLC) analysis. The TLC plate used was a 20 × 20 cm silica gel aluminium TLC plate GF60F254 from Merck. The equipment used in the experiment included general glassware, a CEM^®^ Discover SP MW reactor, a Toledo NewClassic MF analytical balance, a rotary evaporator, a Fischer-Johns melting point apparatus and an Agilent 500 MHz nuclear magnetic resonance (NMR) spectrometer. Calculations using the DFT method were carried out with the following software: Avogadro v. 1.2.0, Chemcraft v. 1.8, Filezilla v. 3.52.2, Termius 7.11.3, MesReNova v. 15.0.0-34764 and ORCA quantum chemistry software v. 4.2.0 [28]. The hardware used is a server computer with the following specifications: a dual Intel Xeon series ES-10 Core processor with 20 threads, 64 GB of RAM, 4 TB of HDD storage and the Linux Ubuntu 20.04 operating system.

### 2.2. Synthesis of ionic liquid 1-decyl-3-methylimidazolium bromide

A total of 5.6 g of 1-methylimidazole and 15 g of 1-bromodecane were placed into a 35 ml one-neck round flask. The reaction mixture was then stirred and irradiated in a CEM^®^ Discover SP MW reactor at 300 W and 50°C for 1 hour. The synthesis results were extracted with *n*-hexane : water = 2 : 1 (v/v) three times and then carried out by vacuum distillation at 160°C and a pressure of 26 cmHg. The synthesized 1-decyl-3-methylimidazolium bromide ([DMIm]Br) ionic liquid was characterized by TLC, ^1^H NMR, and ^13^C NMR spectroscopy.

*1-Decyl-3-Methylimidazolium bromide ([DMIm]Br)*. Yields: 60%. yellow liquid at room temperature. ^1^H NMR δ ppm 0.70 (*t*, 3H); 1.12 (*m*, 14H); 1.75 (*m,* 2H); 4.16 (*t*, 2H); 9.99 (*s*, 1H); 7.58 (*d*, 1H), 7.39 (*d*, 1H); 3.97 (*s,* 3H). ^13^C NMR δ ppm 13.6; 22.1; 25.7; 28.5; 28.7; 28.8; 28.9; 29.8; 31.3; 49.6; 136.5; 123.4; 121.6; 36.2.

### 2.3. Synthesis of 2’-hydroxychalcone derivatives

*Ortho*-hydroxyacetophenone (0.136 g) was added to the solution mixture of an ionic liquid : water with a mass ratio of 0.02 : 1, then 40% (w/v) NaOH in 10% (w/v) aqueous solution of [DMIm]Br was added. Benzaldehyde derivatives, including salicylaldehyde (0.122 g), anisaldehyde (0.136 g) and *ortho*-vanillin (0.152 g), were also added to the mixture. The reaction mixture was irradiated with MWs at 300 W and 80°C for 10 minutes. The reaction mixture was treated with the addition of 37% HCl to obtain a solid product (at pH 2). The solid was filtered, and the pure product was characterized by TLC, melting point analysis, ¹H and ¹³C NMR spectroscopy.

*(E)-2,2’-dihydroxychalcone*. Yields: 65%. Yellow powder. m.p. 140–142 ^o^C. ^1^H NMR δ ppm 7.83 (d, 1H, J = 15.6 Hz (trans)); 8.17 (d, 1H, J = 15.5 Hz (trans)); 6.91-6.98 (m, 3H, J = 8.1–9.6 Hz); 7.58 (d, 1H, J = 7.6 Hz); 7.92 (d, 1 H J = 7.8 Hz); 7.02 (d, 1 H J = 8.2 Hz); 7.49 (t, 1 H J = 7.9 Hz); 7.02 (d, 1H, J = 8.3). ^13^C NMR δ ppm 194.6; 118.7; 141.5; 121.9; 156.4; 116.9; 130.0; 120.3; 130.0; 120.7; 132.3; 120.9; 136.4; 119.0; 163.6.

*(E)-2’-hydroxy-4-methoxychalcone*. Yields: 72%. Yellow powder. m.p. 82–83°C. ^1^H NMR δ ppm 7.63 (d, 1H, J = 15.4 Hz (trans)); 7.91 (d, 1H, J = 15.4 Hz (trans)); 7.62 (d, 1H, J = 8.7 Hz); 6.93 (dd, 1H, J = 9.7 Hz); 3.86 (s, -OCH_3_); 7.92 (d, 1H, J = 1.6 Hz; 6.5 Hz); 7.02 (dd, 1H, J = 8.4 Hz); 7.49 (td, 1H, J = 1.35; 8.4); 6.96 (dd, 1H, J = 2.0; 6.8). ^13^C NMR δ ppm 193.8; 118.7; 145.5; 127.4; 130.7; 114.6; 136.3; 55.6; 118.9; 129.7; 120.2; 136.3; 117.7; 163.7.

*(E)-2,2’-dihydroxy-3-methoxychalcone*. Yields: 81%. Yellow powder. m.p. 182–183°C. ^1^H NMR δ ppm 7.93 (d, 1H, J = 15.5 (trans)); 8.14 (d, 1H, J = 15.6 (trans)); 6.92 (dd, 1H, J = 2.3; 9.8 Hz); 6.91 (t,1H, J = 6 Hz); 7.20 (dd, 1H; J = 1.8;7.2); 3.95 (s, -OCH_3_); 7.95 (dd, 1H, 1.8; 6.4); 7.02 (d, 1H, J = 8.3); 7.49 (td, 1H, J = 1.2; 7.2); 6.94 (d, 1H, J = 8.3). ^13^C NMR δ ppm 194.6; 121.8; 141.1; 120.4; 147.0; 146.3; 120.0; 112.4; 122.4; 56.4; 121.2; 130.0; 118.7; 136.3; 118.9; 163.7.

### 2.4. Computational study

#### 2.4.1. Reactivity study of benzaldehyde derivative precursors with Fukui function

The Fukui function is a theoretical reactivity descriptor formulated within the framework of DFT, as proposed by Parr and Yang [29], to predict sites susceptible to nucleophilic, electrophilic or radical attack in a molecule.

Conceptual DFT introduced the dual descriptor Δf(r) as a tool to simultaneously identify nucleophilic and electrophilic regions by taking the difference between Fukui functions, enabling detailed analysis of local chemical reactivity. This descriptor has been theoretically established and successfully applied to a range of chemical systems, including aromatic compounds and pericyclic reactions to predict regio- and stereoselectivity, offering a unified framework for interpreting reactivity patterns [30–32]. Electrophilic Fukui function (f⁻) descriptors are useful for identifying likely sites of metabolism in aromatic systems. These descriptors often match known metabolic sites on aromatic rings [33].

Analysis of the reactivity towards nucleophiles of three benzaldehyde derivative compounds, namely salicylaldehyde, anisaldehyde and *o*-vanillin, which are precursors in the synthesis of 2’-hydroxychalcone derivative compounds, was done using two different levels of theory: B3LYP [34,35] and ωB97X [36]. Optimization of the neutral structure of each benzaldehyde derivative compound was performed using the KEEPDENS command to generate a density file. After the geometry and density of the neutral charge have been optimized to obtain ρN(r), then ρ*n* + 1(r) (density of the anion) and ρN-1(r) (density of the cation) are determined using the same commands but with a different charge. The condensed Fukui index values (Δf) for each benzaldehyde derivative were calculated using equations (2.1)–(2.5) which simplify the computational process for evaluating local reactivity.


(2.1) $$f^{+}(r)=\rho_{N+1}(r)-\rho_{N}(r)$$

(2.2) $$f^{-}(r)=\rho_{N-}(r)-\rho_{N-1}(r)$$

(2.3) $$Reactivity\;against\;nucleophile:{f^{+}}_{A}={q^{A}}_{N}-{q^{A}}_{N+1}$$

(2.4) $$Reactivity\;against\;electrophile:{f^{-}}_{A}={q^{A}}_{N-1}-{q^{A}}_{N}$$

(2.5) $$\Delta f=f_{A}^{+}-f_{A}^{-};\Delta f>0\;(electrophile);\Delta f<0\;(nucleophile).$$

#### 2.4.2. Mechanism simulation using the density functional theory method

Reaction simulations were carried out on the ionic liquid formation reaction of [DMIm]Br and the cyclization reaction of 2,2’-dihydroxy-3-methoxychalcone to form flavanone. The first step is to optimize all reactants and products of the reaction. The atomic numbering in the reactant and product geometries must be the same, meaning that carbon in the first table must have the same order as in the xyz table in the last table. The transition state is a structure with imaginary frequencies obtained through calculations at the B3LYP level of theory.

#### 2.4.3. Nuclear magnetic resonance simulation using the density functional theory method

NMR calculations were performed based on molecular structures that had first undergone geometry optimization and frequency analysis using the OPT and FREQ commands. These steps ensure that the structure corresponds to a true minimum on the potential energy surface (PES), which is essential for obtaining accurate NMR predictions. Subsequent NMR calculations were performed using the pcSseg-1 basis set within a deuterated chloroform (CDCl₃) solvent, chosen to match the experimental NMR conditions.

The output from these calculations includes the isotopic shielding constants (σ) for each carbon and hydrogen atom, which correspond to the nuclei observed in ¹³C NMR and ¹H NMR, respectively. To convert these shielding constants into chemical shift values (δ, in ppm), a reference shielding value is required. This reference is typically obtained from a separate calculation of the isotopic shielding for tetramethylsilane (TMS), the standard reference compound for both ¹³C and ¹H NMR. The chemical shift for each nucleus is then determined using the equation


(2.6) $$\delta =\sigma_{ref}-\sigma_{cal},$$

where δ is the chemical shift (in ppm), σ_ref_ is the isotopic shielding of the corresponding nucleus in TMS, and σ_cal_ is the isotopic shielding of the same nucleus in the sample compound.

In addition to the calculation of chemical shifts for ¹H NMR, spin–spin coupling constants (J-couplings) between hydrogen atoms were also computed. These values provide important structural and electronic information that complements the chemical shift data. To perform this calculation, the SSAL keyword was included in the ORCA input file. The output from this calculation includes a summary of the isotropic coupling constants, reported in Hertz (Hz). These constants can be directly compared with experimental J values, offering further validation of the predicted molecular structure and conformation.

## 3. Results and discussion

**Figure 1.**
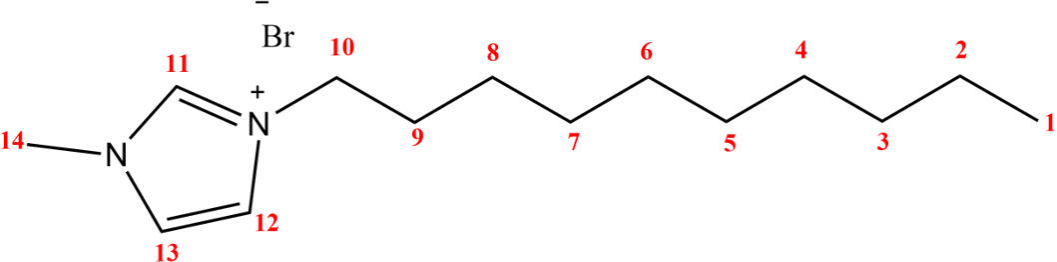
Structure and numbering of [DMIm]Br.

In this research, solvent-free synthesis of [DMIm]Br ionic liquid (figure 1) was carried out using 1-methylimidazole and 1-bromodecane precursors under MW irradiation conditions using the CEM^®^ Discover SP instrument at a temperature of 50°C with an irradiation of 300 W for 1 hour. The resulting product has the physical form of a yellow liquid. The synthesized product was further characterized by ^¹^H NMR spectroscopy at 500 MHz and ¹³C NMR spectroscopy at 125 MHz, using deuterated chloroform (CDCl₃) as the solvent.

**Figure 2.**
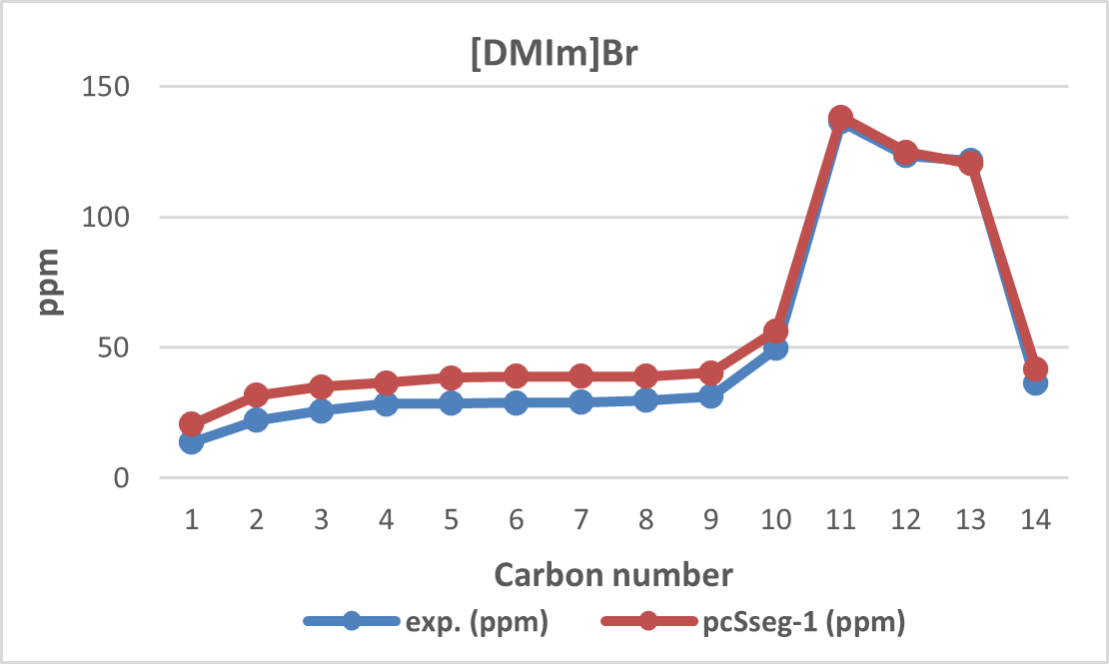
^13^C NMR chemical shift calculated versus experimental of [DMIm]Br.

Computational simulations have also been conducted as part of the study to compare and validate the experimental NMR results. This simulation is carried out using the pcSseg-1 basis. A comparison between the calculated and experimental ¹³C NMR chemical shifts for [DMIm]Br is provided in figure 2.

The ionic liquid ([DMIm]Br) contains 14 carbon atoms, as confirmed by the appearance of 14 carbon signals in the ¹³C NMR spectrum at 125 MHz, observed in both experimental data and computational calculations. Based on its molecular structure, there are three sp² carbon atoms, which originate from the imidazole ring and correspond to carbon atoms C-11, C-12 and C-13. Among these three sp² carbon signals, carbon atom C-11 is identified as the most deshielded. This is because C-11 is located between two highly electronegative nitrogen atoms, resulting in reduced electron density and electron shielding.

The sp³-hybridized carbon atoms are observed within the chemical shift range of 0–90 ppm. The most deshielded signal within this region corresponds to carbon atom C-10, which exhibits the highest downfield shift due to its proximity to the electronegative nitrogen atom. The remaining methylene (–CH₂) groups along the alkyl chain, specifically carbon atoms C-2 to C-8, give rise to signals consistent with typical chemical shifts for sp³-hybridized aliphatic carbons.

**Figure 3.**
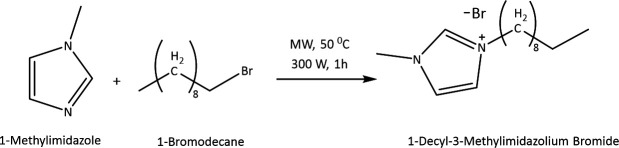
Reaction scheme of [DMIm]Br ionic liquid synthesis.

The synthesis scheme of the ionic liquid [DMIm]Br is shown in figure 3. In theory, if a primary alkyl halide forms a carbocation, it will result in low structural stability, so the reaction mechanism involved in the synthesis of [DMIm]Br ionic liquid is a second-order nucleophilic substitution (S_N_2) mechanism because its tendency to form a carbocation as an intermediate is smaller. The halide atom in 1-bromodecane is Br, which is a good leaving group, so that when an attack by a nucleophile occurs, it will be followed by the breaking of the C-Br bond. To prove this theory, a simulation of the mechanism for the formation of [DMIm]Br ionic liquid was carried out using the DFT method to determine the transition state of the reaction and the accompanying energy. This calculation was carried out using the B3LYP hybrid functional and the DEF2-SVP [37] (a zeta valence with a polarization function) basis set.

**Figure 4.**
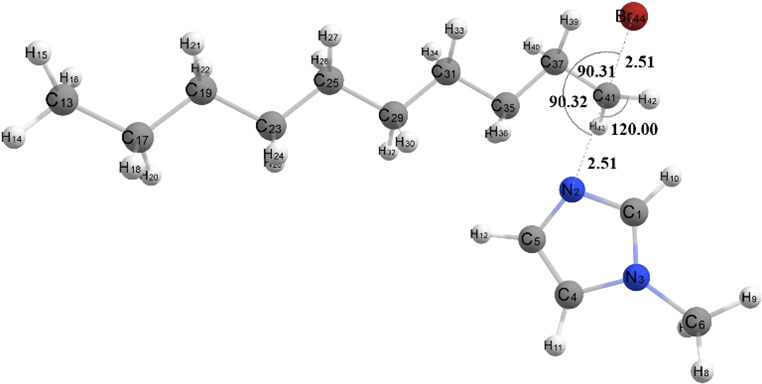
Transition state structure of [DMIm]Br ionic liquid formation reaction.

In figure 4, simulation results show that the bond angles formed in the transition state structure lead to a triangular bipyramidal molecular geometry. The atoms that are in the triangular plane are in an equatorial position, with the angle between the two bonds being 120°. In contrast, the atoms that are above and below the triangular plane are in the axial position. The angle between the axial and equatorial bonds is 90°, while the angle between two axial bonds is 180°. In the transition state structure, it can be seen that the angle between the R-C-Br bond and the R-C-N bond is 90°, whereas the angles between the H-C-H bond and the two H-C-R bonds are 120°. The C-Br and C-N bond distances in the transition state structure are relatively the same; this indicates that in the transition state, N number 2 of 1-methyl imidazole, which acts as a nucleophile, begins to approach C atom number 41, while Br atom number 44 will move further away.

**Figure 5.**
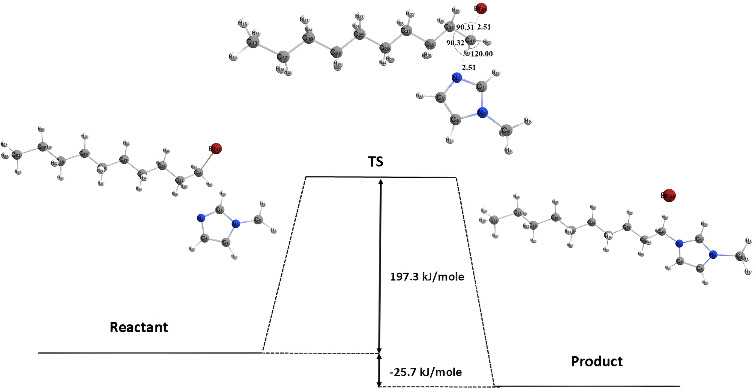
Potential energy surface of [DMIm]Br ionic liquid formation.

**Figure 6.**
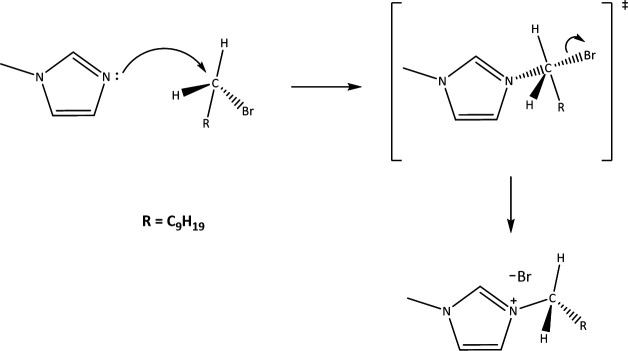
Proposed mechanism of [DMIm]Br formation.

Reaction energy calculations are carried out using the FREQ command and then plotted in the form of a PES, which graphs the energy of reactants as they transition to products to form a certain transition state, as shown in figure 5. Based on the calculations, the activation energy of the reaction to form the ionic liquid [DMIm]Br is 197.3 kJ mol^-1^, with a reaction enthalpy of −25.7 kJ mol^-1^. This indicates that the formation of the ionic liquid [DMIm]Br is an exothermic reaction. Therefore, it is proven that the formation of [DMIm]Br ionic liquid follows the S_N_2 mechanism. The proposed mechanism for the formation of [DMIm]Br ionic liquid is shown in figure 6.

The quaternization reaction was observed to proceed under MW irradiation at a bulk temperature of 50°C, despite its high calculated activation energy of 197 kJ mol^-1^. The distinctive effects of MW-assisted chemistry can explain this. MW irradiation directly interacts with ionic and polar components of the reaction, such as the [DMIm]Br ionic liquid, leading to localized superheating and the formation of ‘hotspots’ where temperatures significantly exceed the bulk temperature [33,34]. This localized energy concentration facilitates the formation of the transition state, effectively lowering the apparent energy barrier at the reaction sites [38,39]. Additionally, MW irradiation introduces non-thermal effects, including dipolar polarization and ionic conduction, which enhance molecular interactions and reduce the kinetic constraints typically associated with high activation energy reactions [38,40,41].

Computational studies [39] have demonstrated that MW irradiation can modify reaction pathways by stabilizing transition states through selective solvation effects and hydrogen bonding networks. These effects are unique to MW irradiation and cannot be replicated by conventional heating, which relies solely on the elevation of bulk temperature. Consequently, the interplay of localized heating, non-thermal activation, and selective energy transfer under MW conditions enables the reaction to proceed efficiently at 50°C, thereby bypassing the thermodynamic barrier that would otherwise require a temperature of at least 200°C with traditional heating methods.

One of the applications of ionic liquids as both a catalyst and solvent is the aldol condensation reaction, also known as the Claisen–Schmidt condensation [42,43]. In this research, the synthesized [DMIm]Br ionic liquid was used as a reaction medium in the synthesis of three 2’-hydroxychalcone derivative compounds from the *o*-hydroxyacetophenone precursor, varying the types of benzaldehyde derivatives: salicylaldehyde, anisaldehyde and *o*-vanillin. These three compounds have been successfully synthesized with yields of 65, 72 and 81%, respectively, and have a physical appearance as a yellow powder.

**Figure 7.**
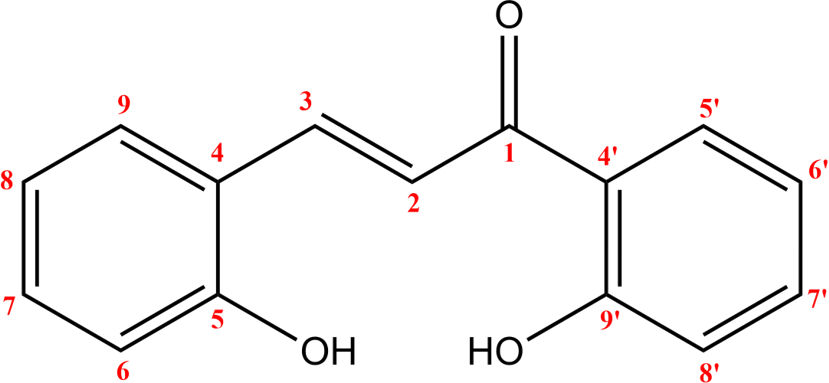
*(E)*-2,2’-dihydroxychalcone structure and numbering.

**Figure 8.**
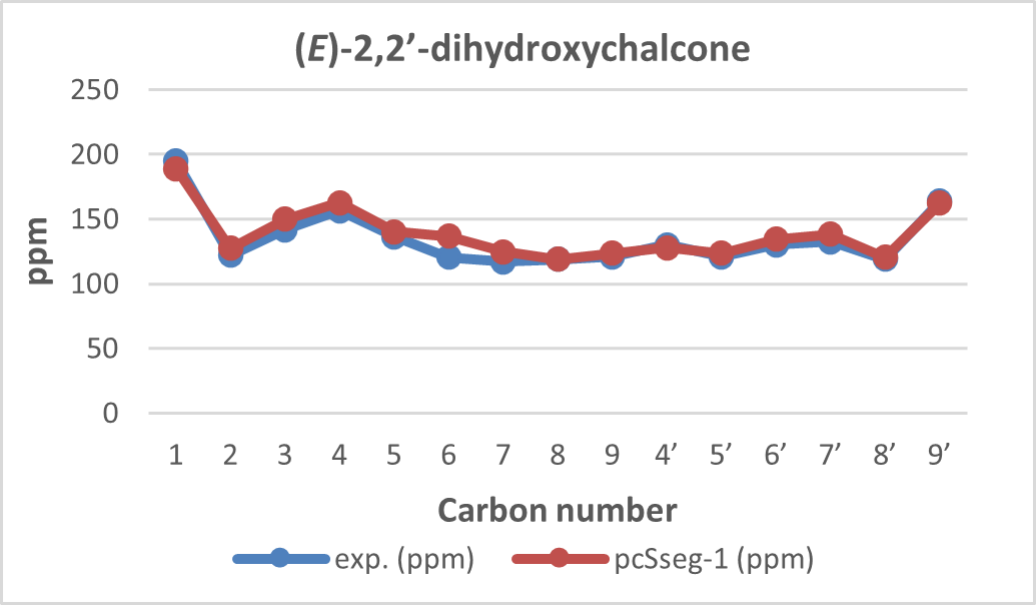
^13^C NMR chemical shift calculated versus experimental of (*E*)-2,2’-dihydroxychalcone.

**Figure 9.**
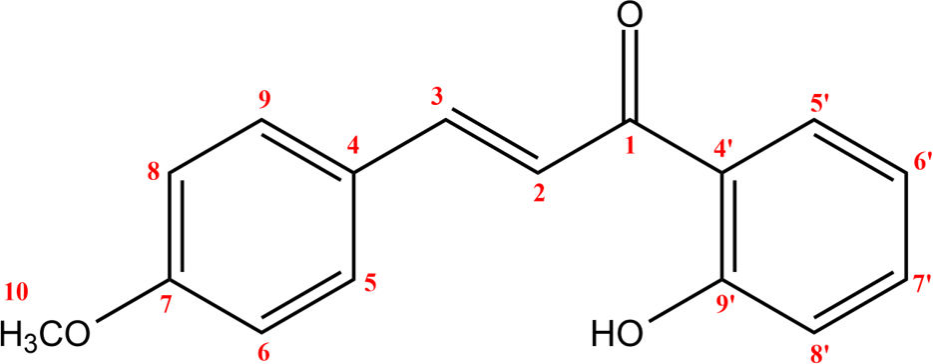
(*E*)-2’-hydroxy-4-methoxychalcone structure and numbering.

**Figure 10.**
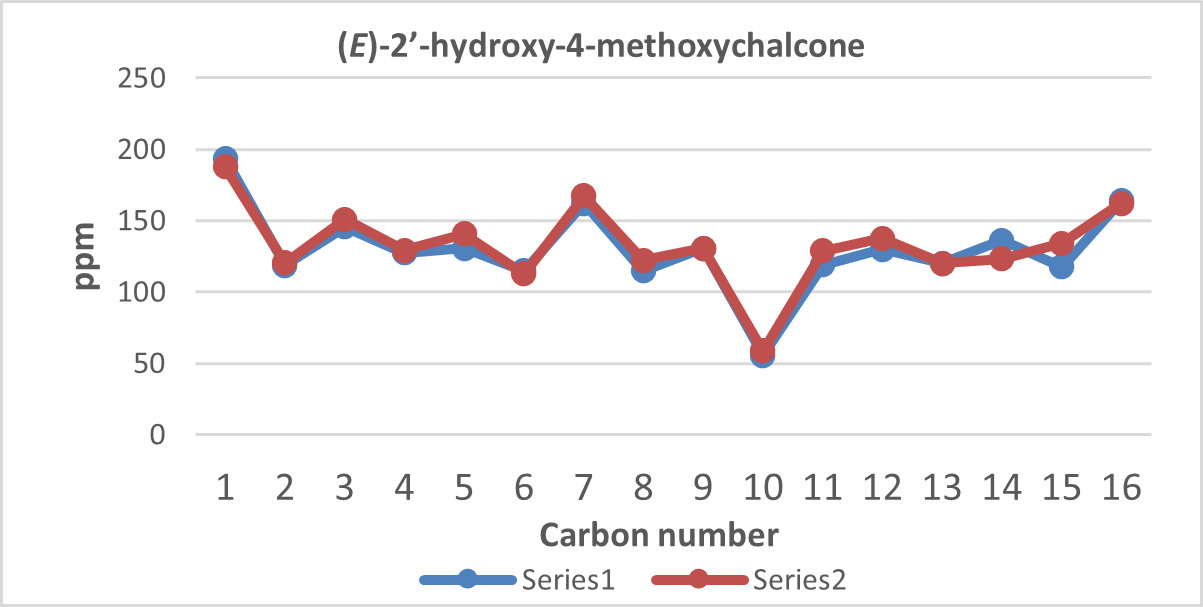
^13^C NMR chemical shift calculated versus experimental of (*E*)-2’-hydroxy-4-methoxychalcone.

**Figure 11.**
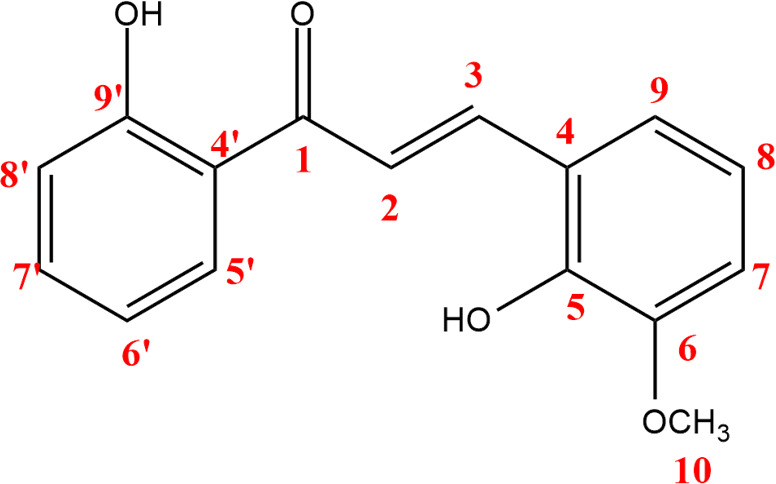
(*E*)-2,2’-dihydroxy-3-methoxychalcone structure and numbering.

**Figure 12.**
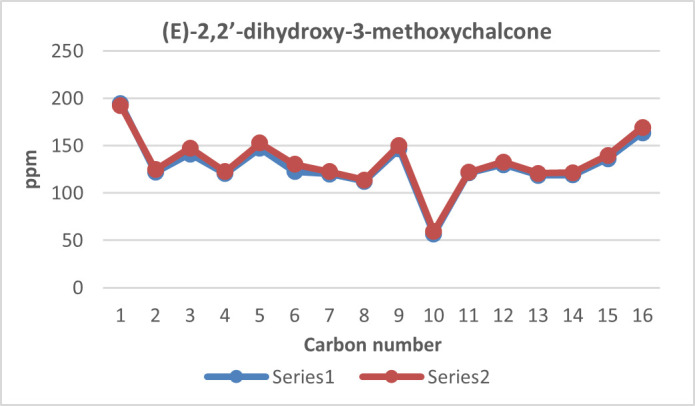
^13^C NMR chemical shift calculated versus experimental of (*E*)-2,2’-dihydroxy-3-methoxychalcone.

The structure of the three synthesized compounds was confirmed using ¹H NMR spectroscopy at 500 MHz and ¹³C NMR spectroscopy at 125 MHz, with deuterated chloroform (CDCl₃) as the solvent. This confirmation was further supported by computational simulation results, as shown in table 1, which demonstrated good agreement with the experimental data presented in figures 7–12.

**Table 1.** Comparison of *J*_H-H_ experimental and calculation of the alkene functional group.

name	interaction	*J*_cal_ (Hz)	*J*_exp_ (Hz)
(*E*)-2,2’-dihydroxychalcone	H12–H14	14.55	15.5 and 15.6
(*E*)-2’-hydroxy-4-methoxychalcone	H12–H14	14.47	15.4
(*E*)-2,2’-dihydroxy-3-methoxychalcone	H22–H23	14.47	15.5 and 15.6

To validate the experimental NMR data, calculations of isotropic spin–spin coupling constants were performed to examine H–H interactions in each compound. These simulations help to confirm the reaction outcome and molecular structure.

**Figure 13.**
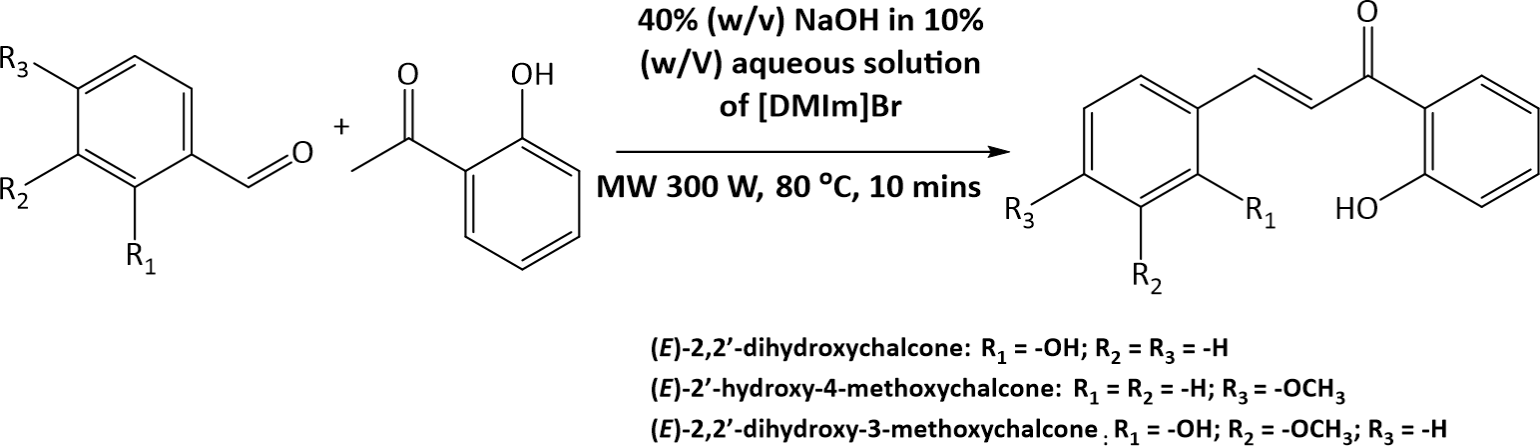
Reaction scheme of 2’-hydroxychalcone derivatives synthesis.

In particular, the coupling constant between alkene protons at the C-α and C-β positions (H-α and H-β) serves as a key indicator. A large ³*J*_H–H_ value typically indicates a trans-alkene configuration, consistent with the expected product of the Claisen–Schmidt reaction (reaction scheme is presented in figure 13). A summary of the H-α and H-β coupling constants for all 2’-hydroxychalcone derivatives is shown in table 1.

The base-catalysed Claisen–Schmidt condensation consists of two reactions. The first reaction is the activation of acetophenone derivative compounds to produce acetophenonate derivative anions. In the second reaction, the acetophenonate anion obtained attacks the carbonyl group of the benzaldehyde derivative to form a chalcone derivative compound [44].

Reactivity studies of benzaldehyde derivatives as electrophiles have been conducted using computational calculations based on the Fukui function, a reactivity index that predicts the process of bond breaking and formation in a series of organic reactions. Through analysis using the Fukui function, it can be determined which group of the three benzaldehyde derivative precursor compounds is most likely to experience an attack by the nucleophile (*o*-hydroxyacetophenone).

**Figure 14.**
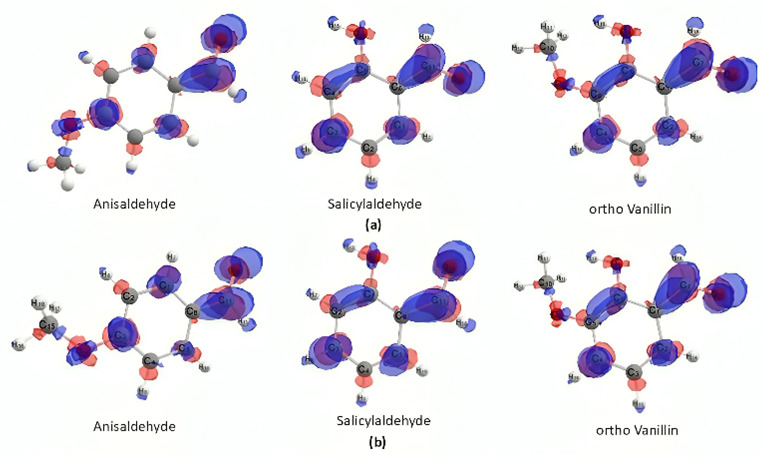
Visualization of structure and electron density of benzaldehyde derivative precursors with hybrid functionals B3LYP (a) and ωB97X (b).

Electron density calculations were performed using two different hybrid functionals: B3LYP and ωB97X. The results of electron density visualization and electron charge calculations for salicylaldehyde, anisaldehyde and *o*-vanillin compounds, using different hybrid functionals, showed a similar trend. The most positive functional group in the three compounds, as calculated, is the C atom of the carbonyl group, characterized by the most dominant positive charge density, as shown in figure 14.

The ∆f is the condensed Fukui index value, where a significant value of ∆f represents reactivity towards nucleophiles. The Fukui function can be used to determine the reactivity of precursors, which influences the ease with which reactions occur and is characterized by the formation of more products for more reactive precursors. The results of computational calculations of the Fukui function, performed using quantitative methods, are then compared with experimental data on the laboratory synthesis of 2’-hydroxychalcone derivative compounds, summarized in table 2.

**Table 2.** Comparison between the Fukui index and the yield of products.

functional hybrid	∆f		
	salicylaldehyde (yield: 65%)	anisaldehyde (yield: 72%)	*o*-vanillin (yield: 81%)
B3LYP	0.10081	0.10859	0.11711
ωB97X	0.10646	0.12233	0.12958

Based on the results of calculations using the Fukui function with both the B3LYP and ωB97X hybrid functionals, it is evident that the benzaldehyde derivative precursor with the most excellent reactivity is *o*-vanillin, followed by anisaldehyde and salicylaldehyde. This is directly proportional to the yield produced through laboratory synthesis. Based on these results, it is proven that the more reactive a benzaldehyde derivative species is, the greater the per cent yield of the 2’-hydroxychalcone derivative obtained. It can be concluded that the calculation of the Fukui index using the DFT method supports the experimental results obtained in the laboratory.

The influence of substituents and their positions in the chalcone synthesis has been reported in several previous studies [45–47]. The three benzaldehyde precursors contain substituents in the form of electron-donating groups, which can cause the aromatic ring to become more negative, resulting in an induced release of electrons from the aromatic ring to the aldehyde functional group. This causes the electron density of the aldehyde functional group to decrease and become more positive, thereby increasing its activity towards nucleophiles.

Apart from that, steric factors also influence the activity of the benzaldehyde group. In this case, the greater the substituent at the *ortho* position, the longer the aldol condensation reaction will take place [48]. This is the reason why anisaldehyde has greater reactivity than salicylaldehyde because the substituents in anisaldehyde are in the para position.

**Figure 15.**
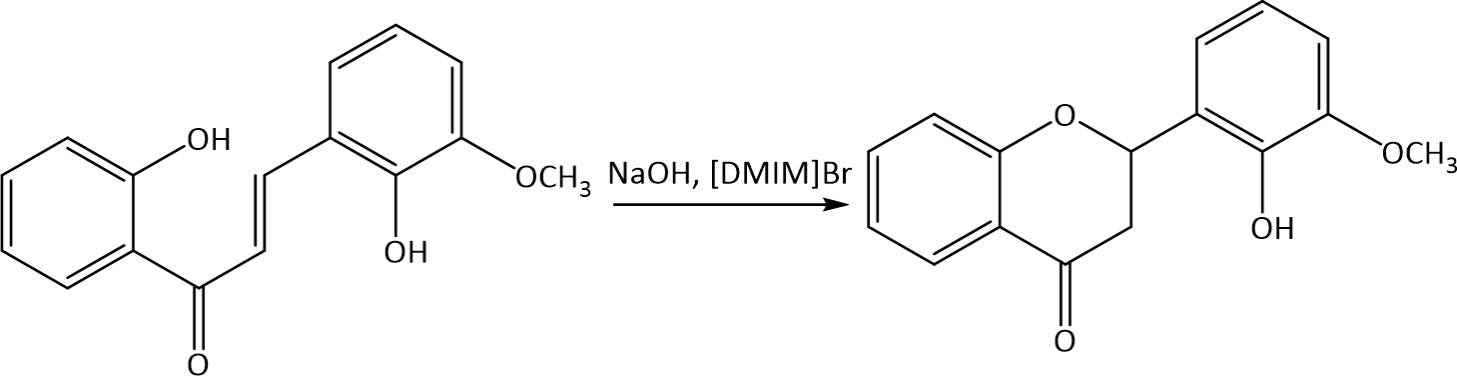
Reaction scheme of 2,2’-dihydroxy-3-methoxychalcone cyclization.

Chalcone is an intermediate of other flavonoid compounds, one of which is a flavanone derivative compound that is obtained through a cyclization reaction under alkaline conditions, as in figure 15. In this study, a reaction mechanism for the formation of flavanone derivative compounds through the cyclization of 2,2’-dihydroxy-3-methoxychalcone in an ionic liquid medium ([DMIm]Br) was investigated using DFT theory.

The use of ionic liquids as catalysts and solvents has been demonstrated to activate various reactions by forming hydrogen bonds, thereby reducing reaction barriers and stabilizing transition states [49]. Ionic liquid [DMIm]Br plays a role in forming hydrogen bonds with oxygen from the carbonyl group so that the 2,2'-dihydroxy-3-methoxychalcone can be more active and easily undergo attack due to the stabilization of the transition state. Additionally, the presence of ionic liquids facilitates the configuration transition of the chalcone to form the *s-trans* isomer, which enables the cyclization reaction to occur more easily.

**Figure 16.**
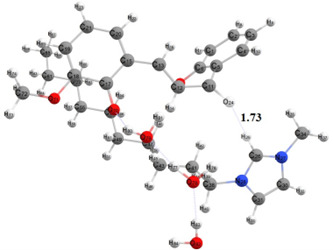
Reactant optimized structure.

The existence of hydrogen bonds in the cyclization stage of 2,2’-dihydroxy-3-methoxychalcone has been proven through optimization calculations of the reactant structure shown in figure 16. It can be seen that the bond distance between the hydrogen atom number 29 in the ionic liquid, which is the most acidic hydrogen because two nitrogen atoms flank it, and the oxygen atom from the carbonyl group in the chalcone is 1.73 Å, which is the bond distance in hydrogen interactions.

**Figure 17.**
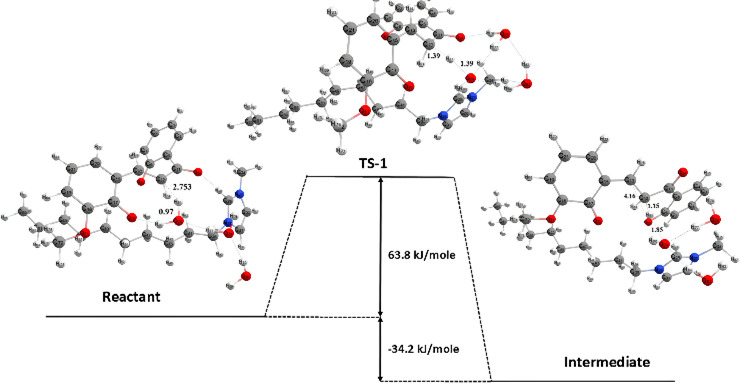
First-stage potential energy surface of the 2,2’-dihydroxy-3-methoxychalcone cyclization reaction.

Simulation using the DFT method revealed that the cyclization reaction of 2,2’-dihydroxy-3-methoxychalcone proceeds through two distinct reaction stages. The results of energy calculations from each stage are made in the form of a PES, which describes the potential energy of a reaction system. Based on the PES from the first stage in figure 17, the activation energy of the reaction in this stage is 63.8 kJ mole^-1^ with a reaction enthalpy of −34.2 kJ mole^-1^, which means that the reaction in this stage is exothermic so that in this stage there is a release of heat from the system to the environment.

At this stage, the transition state structure of the reaction is obtained at a wavenumber of −1330.4 cm^-1^. Through the visualization above, a change in bond distance is observed from the reactant to the transition state and then to the product. Initially, the bond distance between the C-sp2 in the reactant and the hydrogen from water is 2.75 Å, then the hydrogen from water approaches to form a transition state with a bond distance of 1.39 Å. In comparison, the resulting product has a bond distance of 1.15 Å, and a new C-H bond is formed.

**Figure 18.**
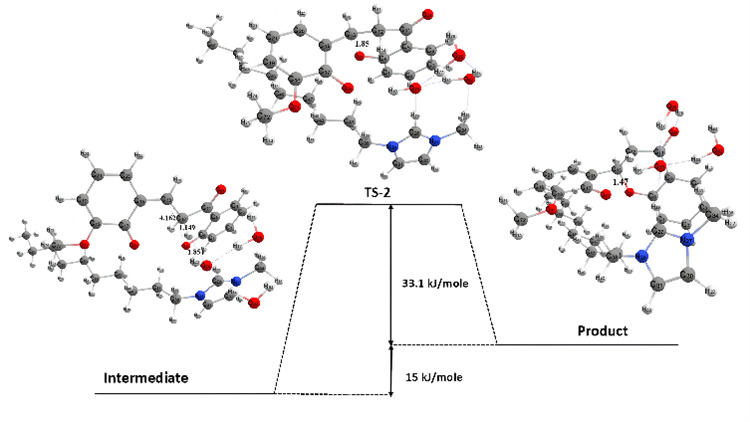
Second-stage potential energy surface of the 2,2’-dihydroxy-3-methoxychalcone cyclization reaction.

The transition state structure of the second stage was obtained at a wavenumber of −236 cm^-1^, with an activation energy of 48.1 kJ mol^-1^ and a reaction enthalpy energy of 15 kJ mol^-1^ (figure 18). It can be seen that the reaction in stage II is endothermic. Based on the visualization results, a change in the bond length between the C and O atoms has occurred, which was originally 4.16 Å and is now 1.85 Å, indicating a transition state. The final product has a bond length of 1.47 Å. The distance between the C and O bonds is getting closer until the bond forms a six-membered ring.

The determining step for the reaction rate (*k*) of the cyclization of 2,2’-dihydroxy-3-methoxychalcone was calculated using the Eyring equation (3.1). The reaction at each stage involves complexes and an elementary reaction, so it can be assumed that the order of each stage is order 1.


(3.1) $$k=\frac{k_{B}.T}{h}e^{\frac{-E_{a}}{RT}},$$

where *k* is the reaction rate constant, *k*_B_ = 1.380649 × 10^−23^ J K^-1^ is Boltzmann’s constant, *h* = 6.62607015 × 10^−34^ J s is Planck’s constant, *T* is the absolute temperature (in K), *E*_a_ is the activation energy and *R* = 8.314 J mole K^-1^ is the universal gas constant.

Based on the results of calculations using the Eyring equation, the stage I reaction rate constant was 4.1 × 10^–1^ s^−1^, while the stage II reaction rate constant was 2.3 × 10^4^ s^−1^. It can be concluded that the stage I reaction is the stage that determines the reaction rate, as it is a slower reaction with an enthalpy of 63.8 kJ mol^−1^.

**Figure 19.**
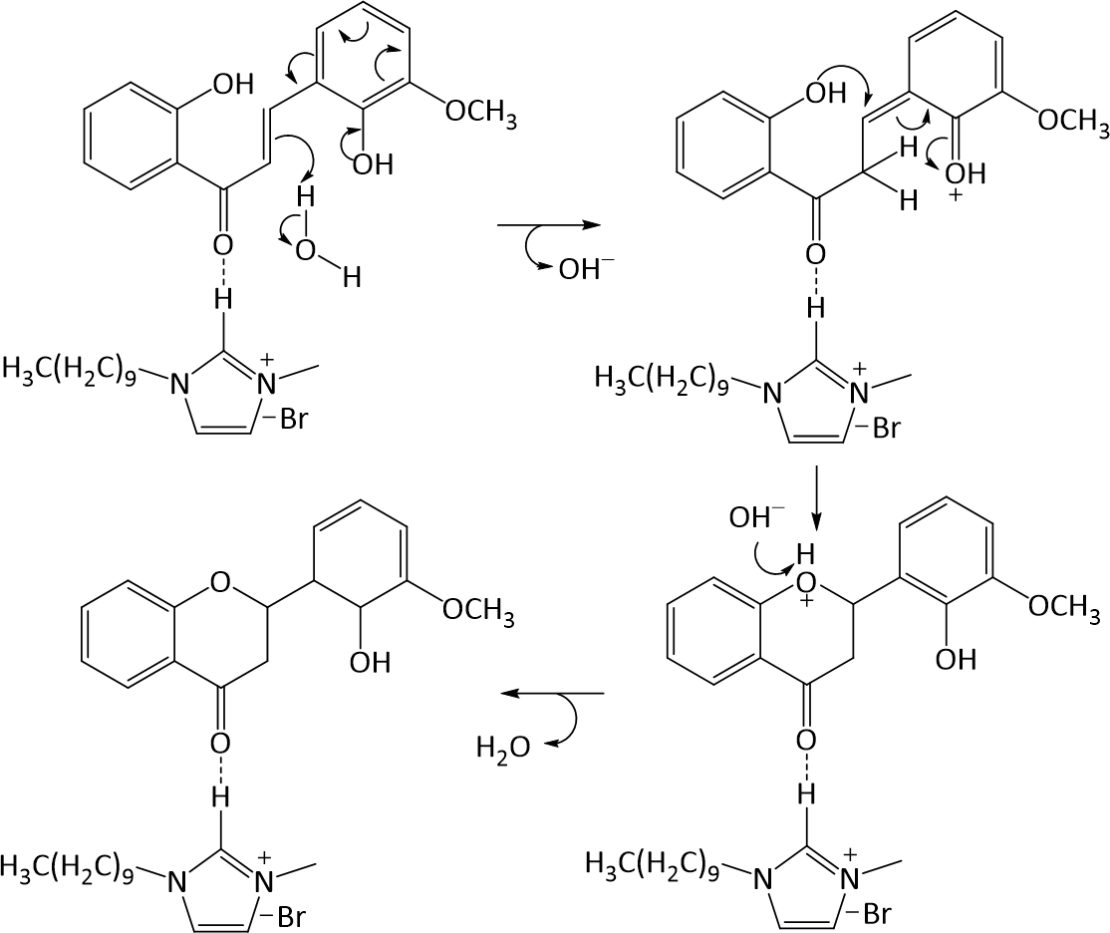
Proposed mechanism of 2,2’-dihydroxy-3-methoxychalcone cyclization.

Imidazolium salts have catalytic effects, particularly in facilitating proton transfer and stabilizing transition states through hydrogen bonding. As shown in figure 19, the NH moiety of the imidazolium salt demonstrates its catalytic function by acting as a hydrogen bond donor, stabilizing anionic intermediates, lowering the activation barrier for cyclization and accelerating ring closure steps. This dual role of hydrogen bonding and proton transfer aligns with studies that reported similar catalytic behaviours in NH-functionalized ionic liquids [50–52]. These findings reinforce the proposed mechanism, highlighting the distinctive combination of catalytic and solvent properties characteristic of imidazolium-based ionic liquids.

## 4. Conclusion

Ionic liquid [DMIm]Br has been successfully synthesized using MW irradiation at a temperature of 50°C, power 300 W, for 1 hour and then applied as a reaction medium in the synthesis of three derivative compounds of 2'-hydroxychalcone. The compounds 2,2'-dihydroxychalcone, 2’-hydroxy-4-methoxychalcone and 2,2’-dihydroxy-3-methoxychalcone have been successfully synthesized using MW irradiation at a temperature of 80°C, power 300 W, for 10 minutes with a yield of 65, 72 and 81%, respectively. All synthesized compounds were confirmed by ¹³C and ¹H NMR spectroscopy, with computational data in strong agreement with the experimental results. Fukui index calculations, performed using both B3LYP and ωB97X hybrid functionals, were in agreement with experimental results, which demonstrated that the yield obtained was greatest, consistent with the reactivity of the benzaldehyde derivative precursor. The reactivity of *o*-vanillin was the greatest, followed by anisaldehyde and salicylaldehyde. A study of the reaction mechanism with the functional hybrid B3LYP proves that the formation of [DMIm]Br ionic liquid, following the S_N_2 reaction mechanism, produces an activation energy of 197.3 kJ mol^−1^. Based on simulations carried out using the DFT method, the cyclization mechanism of the 2,2’-dihydroxy-3-methoxychalcone compound involves two reaction stages, with the first stage being the rate-determining step, characterized by an activation energy of 63.8 kJ mol^−1^.

## Data Availability

The data can be accessed in the following link [53]. Supplementary material is available online [54].
